# Role of Nitric Oxide-Derived Metabolites in Reactions of Methylglyoxal with Lysine and Lysine-Rich Protein Leghemoglobin

**DOI:** 10.3390/ijms24010168

**Published:** 2022-12-22

**Authors:** Konstantin B. Shumaev, Olga V. Kosmachevskaya, Elvira I. Nasybullina, Enno K. Ruuge, Alexey F. Topunov

**Affiliations:** 1 Bach Institute of Biochemistry, Research Center of Biotechnology, Russian Academy of Sciences, 119071 Moscow, Russia; 2 E.I. Chazov National Medical Research Center of Cardiology, 121552 Moscow, Russia

**Keywords:** lysine, leghemoglobin, nitric oxide, methylglyoxal, carbonyl stress, Maillard reaction, electron paramagnetic resonance

## Abstract

Carbonyl stress occurs when reactive carbonyl compounds (RCC), such as reducing sugars, dicarbonyls etc., accumulate in the organism. The interaction of RCC carbonyl groups with amino groups of molecules is called the Maillard reaction. One of the most active RCCs is α-dicarbonyl methylglyoxal (MG) that modifies biomolecules forming non-enzymatic glycation products. Organic free radicals are formed in the reaction between MG and lysine or Nα-acetyllysine. S-nitrosothiols and nitric oxide (^•^NO) donor PAPA NONOate increased the yield of organic free radical intermediates, while other ^•^NO-derived metabolites, namely, nitroxyl anion and dinitrosyl iron complexes (DNICs) decreased it. At the late stages of the Maillard reaction, S-nitrosoglutathione (GSNO) also inhibited the formation of glycation end products (AGEs). The formation of a new type of DNICs, bound with Maillard reaction products, was found. The results obtained were used to explain the glycation features of legume hemoglobin—leghemoglobin (Lb), which is a lysine-rich protein. In Lb, lysine residues can form fluorescent cross-linked AGEs, and ^•^NO-derived metabolites slow down their formation. The knowledge of these processes can be used to increase the stability of Lb. It can help in better understanding the impact of stress factors on legume plants and contribute to the production of recombinant Lb for biotechnology.

## 1. Introduction

**Figure 1:**
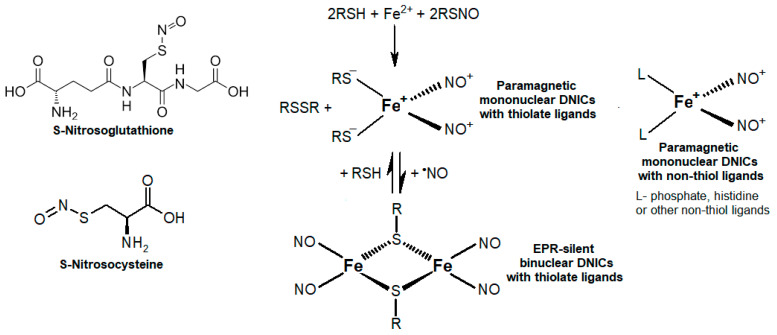
Important physiological ^•^NO derivatives. On the left—S-nitrosothiols (RSNOs), on the right—dinitrosyl iron complexes (DNICs) with thiol and non-thiol ligands.

Nitric oxide (^•^NO) holds a unique position among the palette of all free radicals formed in living systems; it is involved in many signaling pathways and fulfills other essential functions in every life kingdom [1,2,3]. Among many others, the list of key ^•^NO-derived metabolites includes S-nitrosothiols (RSNOs) and dinitrosyl iron complexes (DNICs), which are formed in vitro and in vivo with heme and a pool of chelatable iron [3,4,5]. These metabolites determine ^•^NO’s functioning both as a signaling and regulatory molecule [2,3,5]. Figure 1 shows the main low-molecular-weight RSNOs and DNICs with thiol and non-thiol ligands.

Various ^•^NO-derived metabolites often contribute to the so-called nitrosative stress, which is frequently associated with the well-known oxidative type of stress. However, apart from those types of stresses, there is also a less known, yet no less important, carbonyl stress. It occurs in living organisms in the course of accumulating reactive carbonyl compounds (RCCs), including reducing sugars and dicarbonyls such as 3-deoxyglucasone, methylglyoxal (MG) and glyoxal [1,6,7,8,9]. In humans, carbonyl stress develops in diabetic hyperglycemia and a number of other pathologies [6,7,8,9,10]. RCCs interact with both low-molecular biomolecules and biopolymers, leading to their modification and occasional inactivation [6,7,8,11,12]. The main mechanism of such a modification is the interaction of free amino groups with RCCs (the Maillard reaction) [6,7,8,9,10,11]. Thus, glycation arises from the reaction of amino acids (most often lysine, arginine and cysteine) with reducing sugars and their auto-oxidation products. In addition, RCCs react with guanidine, thiol and imidazole groups of arginine, cysteine, and histidine, respectively [6,7,8,11]. The non-enzymatic glycation of protein amino acid residues has several stages and ends with the formation of advanced glycation end-products (AGEs), e.g., when lysine and arginine residues react with reducing sugars, Amadori and Heyns compounds are formed. They are easily oxidized, resulting in stable glycation end-products (AGEs).

Carbonyl stress is accompanied by an increased production of reactive oxygen (ROS) and reactive nitrogen (RNS) species, leading to a strong oxidizer, peroxynitrite, being formed in the ^•^NO reaction with superoxide radicals [7,12,13,14].

Using spin traps in electron paramagnetic resonance (EPR) spectroscopy, it has been shown that free radicals are formed when MG is oxidized by peroxynitrite [15]. The mentioned EPR spectroscopy is one of the main methods used to study the processes occurring under oxidative and nitrosative stresses. First of all, it applies to the reactions of the free radical oxidation of biological molecules under ROS and RNS action. In the present work we actively used this method as well.

Free radical intermediates also occur in non-enzymatic glycation reactions. The free radicals of melanoidin formed in the glucose reaction with glycine were registered back in 1965 by Mitsuda et al. [16]. When MG interacts with amino acids and proteins, superoxide anion radicals (O_2_^•−^) and organic free radicals are formed; the latter were recorded directly by EPR spectroscopy [12,17,18]. The first compound of the kind—MG cis anion radical (2,5-dimethyl-l,4-semidion)—was registered in the reaction of methylamine with MG [19]. Asahi et al. measured the production of free radical intermediates during the non-enzymatic albumin glycation by ribose using a α-phenyl-tert-butylnitrone spin trap [20]. Of note, the formation of AGEs (pentosidines) in these reactions was inhibited by ^•^NO donors. In Mitsuda’s aforementioned work, NO also reduced the yield of Maillard reaction products [16].

Lysine is the most-often glycated amino acid in proteins, so many of the registered AGEs are formed on the basis of lysine or cross-linked pairs such as Lys–Lys and Lys–Arg. Therefore, as glycation targets, we used lysine or Nα-acetyl-lysine as a comparative analog of lysine residue in proteins.

Studying the role of ^•^NO-derived metabolites in MG’s action on lysine is also important because it allows to gain an insight into the effect of carbonyl stress on the hemoglobin (Hb) of legume plants, leghemoglobin (Lb). Lb functioning is necessary for a symbiotic nitrogen-fixing system, consisting of legume plants–nodule bacteria (Rhizobia). Due to its high affinity for oxygen, Lb maintains oxygen conditions in nodules, which is optimal for the nitrogen-fixation process. It performs the function of a buffer and oxygen carrier to nitrogen-fixing bacteroids—a symbiotic form of Rhizobia [21,22,23]. Additionally, it has also been considered as a component to produce so-called “vegetable meat” [24,25]. Thus, the effect of various stress factors on Lb functioning has become crucial for biotechnology applications as well.

Lb is a lysine-rich protein. The analysis of various Lb amino acid sequences showed that lysine accounts for ~10% of all amino acids in Lb [26], which is about two times higher than the average lysine content in proteins [27]. There are also two arginine residues per Lb molecule, except for lupin Lb, which is drastically different from the other ones [26]. It should be noted that the proteome analysis of bean nodules (*Phaseolus vulgaris*) revealed that lysine was the amino acid most susceptible to glycation and carbonylation [28].

Lb is a very specific Hb because, unlike many other types of hemoglobin, it does not contain cysteines [26]. As the cysteine residues in proteins are among the most active and can interact with various reactive compounds, other amino acids, including lysine, have to perform their role in Lb. Cysteines are also the most common amino acids that become ligands for protein-bound DNICs, including the Hb-bound ones [29,30]. Then, in the absence of cysteine, lysine residues can become such ligands. Therefore, scrutinizing lysine reactivity as free amino acids can help to clarify the mechanisms of RNS and MG interaction with Lb.

Thus, this study aimed to investigate the role of ^•^NO-derived metabolites in MG reactions with lysine, whose residues are the main target for MG in proteins. We also hope to apply the results of these studies to explain the processes of MG’s interaction with Lb—a lysine-rich protein. This contributes to the elucidation of interaction mechanisms between reactive small molecules—including ROS, RNS and RCC—during oxidative, nitrosative and carbonyl stresses.

## 2. Results

### 2.1. Effect of S-Nitrosothiols on the Formation of Free Radicals in the Maillard Reaction

**Figure 2:**
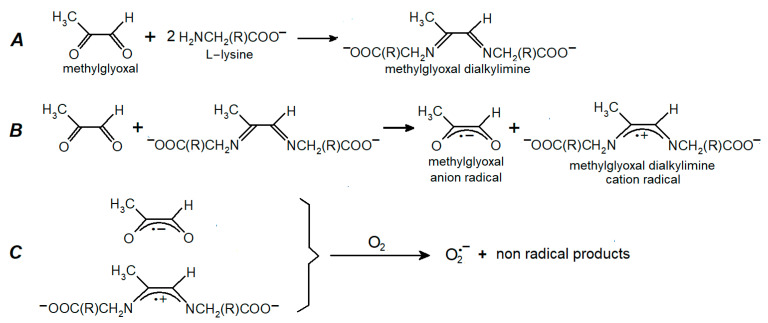
The scheme of free radical intermediate formation during MG’s interaction with lysine. (**A**) The formation of the MG Schiff base with lysine (MG dialkilimine). (**B**) The one-electron oxidation of dialkylimine by MG that forms MG anion radicals (semidion) and Schiff base cation radicals. (**C**) The production of superoxide anion radicals (O_2_^•−^) as a result of the one–electron oxidation of MG and dialkylamine free radicals by O_2_.

**Figure 3:**
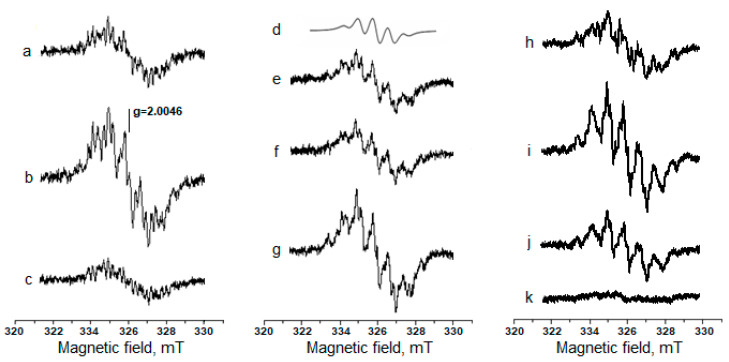
EPR spectra of MG anion radicals and dialkylimine cation radicals formed in the Maillard reaction. The reaction mixture: 0.2 M K, Na-phosphate buffer (pH 8.0), 160 mM MG, and 160 mM (spectra *a–c*) or 80 mM (spectra *e–f*) lysine, or 160 mM Nα-acetyllysine (spectra *h–k*). The simulation of the EPR spectra of MG cis-anion radicals (*d*). During various experiments, 4 mM GSNO (b,g), 4 mM Angeli’s salt (*c*); 1 mM DTPA (*f, h–k*); 4 mM CysNO (*i*, *j*) or 1.25 mM DNICs with cysteine ligands (*j*) were added to the reaction mixture. EPR spectra were recorded 10.5 min after mixing the components with nitrogen purging (*a–j*) or with aeration during the last 4 min of incubation (*k*). High-frequency modulation amplitude—0.2 mT.

One of the main targets of MG and other RCC is lysine residues in the protein molecules [6,8,11]. Therefore, we used a system containing MG and lysine (the L-lysine was used in the experiments) to simulate carbonyl stress. We previously applied this model system to study the production of free radicals in the Maillard reaction [12]. Of note, in some experiments Nα-acetyllysine was used as an analogue to the lysine residue in proteins. It is known that the reaction of MG carbonyl groups with amino groups of biomolecules produces cross-linked Schiff bases (dialkylimines). The formation of dialkylimine with two lysine residues leads to cross-linking in proteins [6,7,11,18]. If dialkilimine interacts with another MG molecule, MG anion radicals and dialkilimine cation radicals are formed [12]. We have proposed a scheme summarizing these data (Figure 2). Figure 3 shows the EPR spectra of MG anion radicals (MG^•−^) and dialkylimine cation radicals recorded after the passage of gaseous nitrogen through the reaction mixture containing MG and lysine or Nα-acetyllysine.

The molar ratios of MG and lysine 1:1 (*a-c* spectra) or 2:1 (*e-g* spectra) were used, and at the latter, the input of a five-component EPR signal that was characteristic of MG semidion was observed. An increase in the semidion level was also observed in a mixture containing MG and Nα-acetyllysine in a 1:1 ratio. Since one of the Nα-acetyllysine amino groups was blocked, the molar ratio of MG carbonyl groups and free amino groups in this system was the same as it was with MG and lysine, i.e., 2:1.

**Figure 4:**
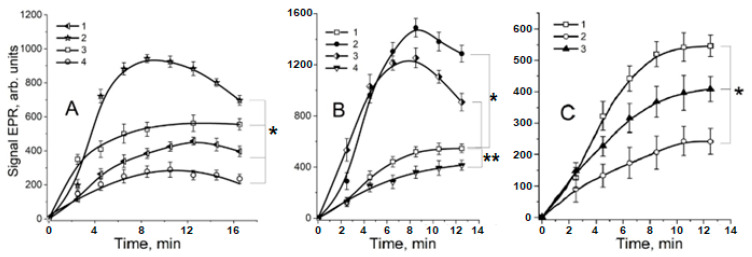
Kinetics of the formation of organic free radicals in the Maillard reaction. A—formation of free radical intermediates in the MG reaction with lysine. Reaction mixture: (1) − MG + lysine, both 100 mM; (2) − (1) + 4 mM S-nitrosoglutathione (GSNO); (3) − (1) + 4.8 mM PAPA NONOate; (4) − (1) + 4 mM Angeli’s salt. B—formation of free radical intermediates in the MG reaction with Nα-acetyllysine. (1) − 160 mm MG + 160 mM Nα-acetyllysine; (2) − (1) + 4 mM GSNO; (3) − (1) + 4 mM S-nitrosocysteine (CysNO); (4) − (1) + 1 mM DNICs containing reduced glutathione (GSH). C—inhibition of free radical production by nitroxyl anion and GSH. (1)—the same as B1; (2) − (1) + 1 mM Angeli’s salt; (3) − (1) + 4 mM GSH. EPR spectra were recorded 2.5 min after mixing the components. Modulation amplitude—0.4 mT. Results are depicted as the mean with S.E.M. of three independent experiments (*n* = 3). (**A**) Two-way ANOVA (* *p* ≤ 0.05 for all point after 4 min). (**B**) Student *t*-test (* *p* ≤ 0.05 for all point after 2 min; ** *p* ≤ 0.05 for all point after 4 min). (**C**) Two-way ANOVA (* *p* ≤ 0.05 for all point after 4 min).

The data presented in Figure 3 and Figure 4 show that such RSNOs as S-nitrosoglutathione (GSNO) and S-nitrosocysteine (CysNO) significantly increased the yield of organic free radicals in the MG reaction with lysine or Nα-acetyllysine. The kinetics of these free radical formations demonstrate that their maximum level in the presence of RSNOs was detected after an 8 min incubation of the reaction mixture (Figure 4A, curve 2 and Figure 4B, curves 2, 3).

We cannot rule out the possibility that ions of contaminant iron are involved in the free radical reactions during non-enzymatic glycation. Nevertheless, adding the iron chelator DTPA to the reaction medium practically did not affect the production of EPR-registered free radicals (Figure 3 and Figure 4). It was previously shown that the free radical organic intermediates of the Maillard reaction are oxidized by molecular oxygen to form a superoxide anion radical (O_2_^•−^) and non-radical products [12,18]. Thus, with an increase of O_2_ partial pressure, a sharp decrease in the level of the free radicals of MG and dialkylimine was observed [Figure 3, spectrum *k*]. So, the registration of these free radicals was carried out under anaerobic conditions (with nitrogen purging), which also excluded the formation of peroxynitrite (ONOO^−^) in a diffusely controlled superoxide reaction with nitric oxide:

^•^NO + O_2_^•−^ → ONOO^−^. (1)

Thus, the possible effect of ONOO^−^ on non-enzymatic lysine glycation by MG was prevented [15].

**Figure 5:**
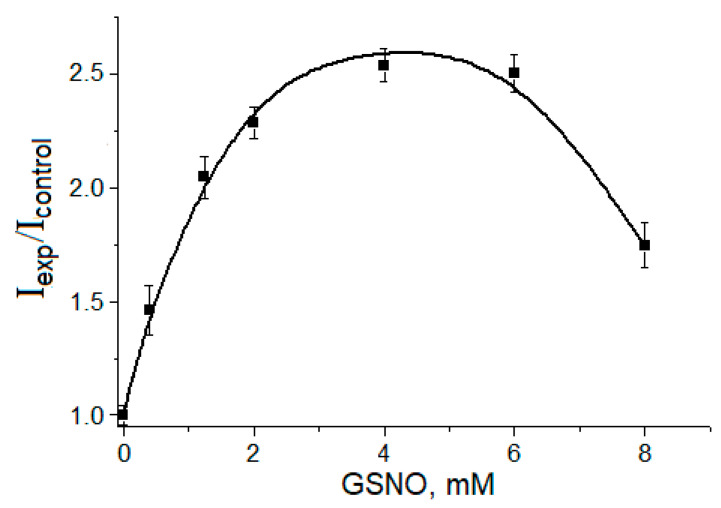
Levels of MG anion radicals and dialkylimine cation radicals depending on GSNO concentration. Reaction mixture: 0.2 M K,Na-phosphate buffer (pH 8.0), 160 mM lysine, 160 mM MG and GSNO. Incubation with constant nitrogen purging. EPR signals were recorded 8.5 min after mixing the components. I_exp_/I_control_—the ratio of the EPR signal intensity in the samples with GSNO to the signal in the control sample (without GSNO).

The level of the organic free radical intermediates of the Maillard reaction was shown to be dependent on the GSNO concentration (Figure 5). The dependence had a bell-shaped character, and the greatest yield of these intermediates was at the GSNO concentration equal to 4–6 mM.

**Figure 6:**
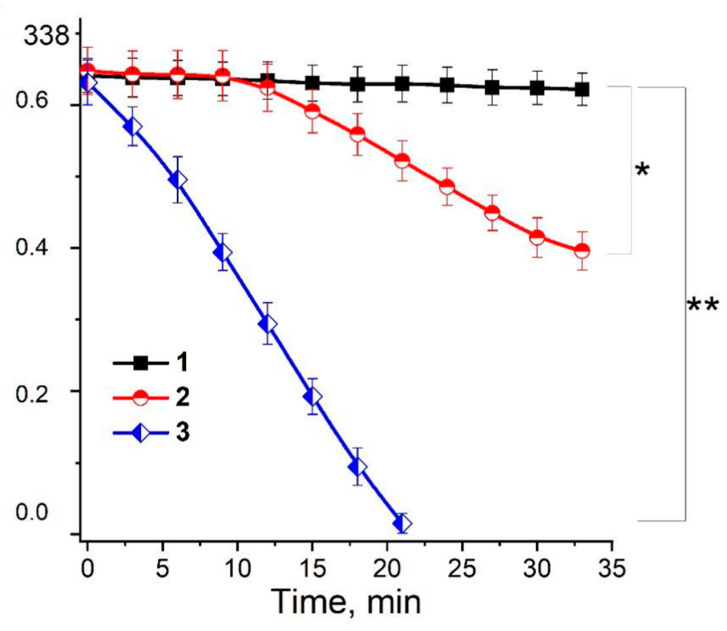
Kinetics of GSNO degradation. Reaction mixture: 0.2 M K,Na-phosphate buffer (pH 8.0) and 0.75 mM GSNO (1); (1) + lysine and MG (both 100 mM) (2); (2) + 0.1 mM CuSO4 (3). Results are depicted as the mean with S.E.M. of three independent experiments (*n* = 3). Student *t*-test (* *p* ≤ 0.05 for all point after 15 min, ** *p* ≤ 0.05 for all point after 5 min).

It is possible that the production of the free radical intermediates in the Maillard reaction can be stimulated by both RSNO and ^•^NO formed during RSNO decay. Indeed, the NO donor PAPA NONOate also increased the yield of the free radicals of MG and dialkylimine, although less effectively than RSNOs (Figure 4A, curve 3). In addition, we found that in a reaction medium containing lysine and MG, GSNO is rapidly destroyed (Figure 6). The decay of this RSNO is significantly stimulated by adding Cu^2+^ ions, which are most probably reduced by the products of the Maillard reaction to Cu^1+^ (Figure 6, curve 3). It is known that, in the presence of a reducing agent, copper ions catalyze the destruction of GSNO, according to the following reaction [31]:

Cu^1+^ + GSNO → Cu^2+^ + GS^−^+ ^•^NO (2)

At the same time, during the one-electron reduction of RSNOs, the anion radical (RSNO^•−^) is formed [31,32]:

RSNO + *e^−^* → RSNO^•−^ → RS^−^ + ^•^NO. (3)

We suppose that the detected effects can be explained by the fact that RSNOs and ^•^NO are the mediators of dialkylimine oxidation by MG (Figure 2B). This leads to an increase in the amount of free radicals formed in this reaction. According to this assumption, RSNO and NO oxidize the cross-linked Schiff base (lysine dialkylamine with MG) to its cation radical in the following reactions:

dialkylimin + RSNO → dialkylimin^•+^ + RSNO^•−^, (4)

dialkylimin + ^•^NO ←→ dialkylimin^•+^ + NO^−^. (5)

Moreover, it could be assumed that RSNO^•−^ and nitroxyl anions (NO^−^) reduce MG to semidion (MG^•−^):

MG + RSNO^•−^ → MG^•−^ + RSNO, (6)

MG + NO^−^ → MG^•−^ + ^•^NO. (7)

Nevertheless, the donor of the nitroxyl anion, Angeli’s salt, reduces the level of organic free radical intermediates produced in the reactions of MG with lysine and Nα-acetyllysine (Figure 3 and Figure 4). Consequently, NO^−^ is unlikely to interact with MG, i.e., reaction 7 is not implemented. However, NO^−^ and its protonated form, nitroxyl (HNO), are probably capable of reducing MG anion radicals (MG^•−^) with the formation of non–radical products. A similar reaction was proposed in the article [12] to explain the MG^•−^ interaction with superoxide radical. It is also known that HNO can reduce lipid free radicals, which is associated with its antioxidant effect [33,34].

On the other hand, the products of NO interaction with intermediates of the Maillard reaction can serve as mediators in the formation of dialkilimine and MG free radicals. In particular, such products can be the N-nitroso derivatives of Amadori products, which are the result of Schiff bases’ transformation in the Maillard reaction [35,36]. It was also shown that ^•^NO is able to attack the double bond in Schiff bases with the formation of reactive compounds, including free radicals [37].

### 2.2. The Role of Dinitrosyl Iron Complexes in the Reaction of Non-Enzymatic Glycation of Lysine by Methylglyoxal

Currently, the biological functions of thiol-containing DNICs are the best-studied, among others [5,29,38,39,40]. Their formation can be ensured by RSNOs [39,41,42]. In a system modeling carbonyl stress, DNICs containing reduced glutathione (GSH) and cysteine decreased the content of EPR-detected free radicals (Figure 3, spectrum j and Figure 4B, curve 4). When cysteine-containing DNICs and CysNO were jointly added to the reaction mixture in similar concentrations, the effect of CysNO was completely inhibited (Figure 3). We have previously shown that MG can modify the cysteine ligands of DNICs [43]. Thiols, e.g., GSH, react with MG, forming hemithioacetals. GSH, apart from the anti-glycating effect, displays remarkable antioxidant properties and effectively intercepts free radicals [1,10,13,43]. In our experiments, GSH decreased the amount of the free radicals of MG and dialkylimine (Figure 4C, curve 3). It can be assumed that the accumulation of thiols formed in reaction 3 decreased the level of free radical intermediates at high GSNO concentrations (Figure 5). Thus, the antiradical action of GSH- and cysteine-containing DNICs may be associated with their thiol ligands, whereas NO, as part of DNICs, most probably does not take part in reactions 4–6.

Along with thiols, DNIC ligands can be represented by phosphate anions, protein histidine residues or carnosine dipeptide [29,40,42,44], as well as the products of cysteine glycation by MG [43]. It is known that the redox-active ions of transition metals catalyze the glycation and glycooxidation of proteins [7,11,12]. At the same time, some products of the Maillard reaction chelate these metals [45,46]. In addition, synthetic Schiff bases form complexes with transition metals and ^•^NO [47,48]. We investigated whether it was possible for DNICs to be formed with the products of the non-enzymatic glycation of lysine, enhanced by the presence of MG.

**Figure 7:**
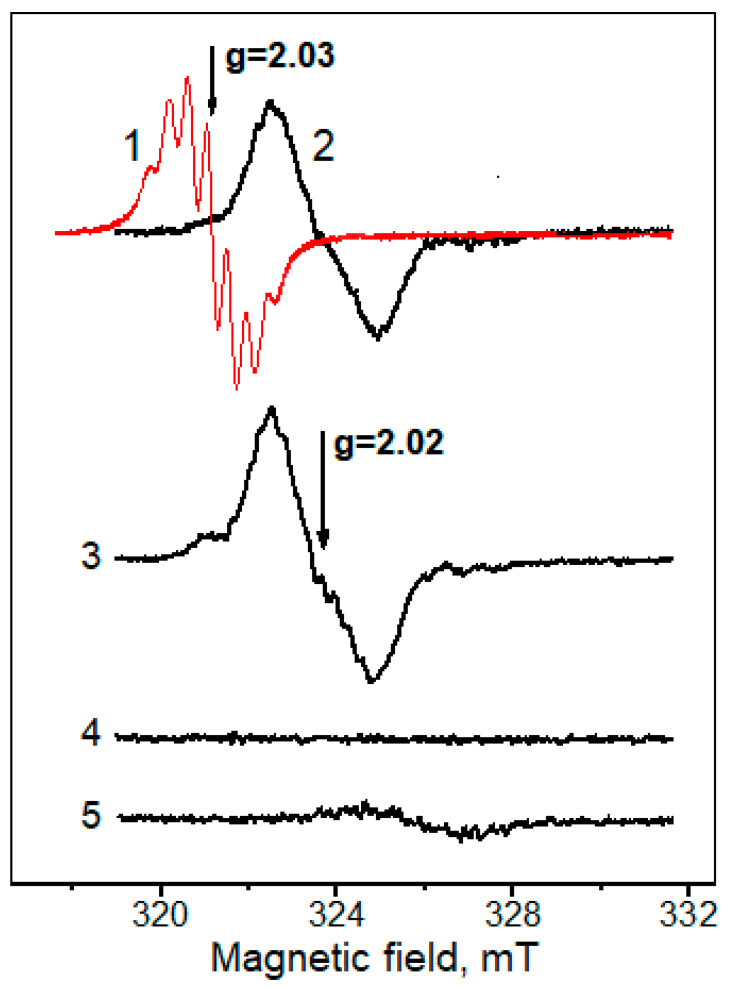
Formation of the new type of DNICs bound with lysine and modified with MG. (1)—reaction mixture: 0.2 M PBS (pH 8.0), 160 mM MG and 1 mM DNIC-PO_4_^−^; (2)—(1) + 160 mM lysine; (3)—the same as (2), but instead of DNIC-PO_4_^−^, 0.4 mM FeSO_4_ and 8 mM PAPA NONOate were added. (4)—160 mM lysine + 0.4 mM FeSO4 + 8 mM PAPA NONOate. (5)—the same as (2) + 1 mM bathophenanthroline added after 5 min. EPR spectra were recorded 2.5 min after mixing the components at nitrogen purging. The amplitude of the high-frequency modulation—0.1 mT.

In our experiments, when unstable phosphate DNICs were added to the reaction mixture containing lysine and MG, a new EPR signal with a g-factor of 2.02 was registered (Figure 7, spectrum 2). A similar result was obtained after Fe^2+^ ions and PAPA NONOate as an ^•^NO donor were added to the reaction mixture (Figure 7, spectrum 3). It can be assumed that in the first case, phosphate DNICs are donors of the Fe(NO)_2_ group, transferred to the new ligands that are formed in the Maillard reaction. In the second case, the formation of a new type of paramagnetic DNIC apparently occurs, with ^•^NO and iron ions taking part in the reaction, as proposed in the article [39]. The modified image of this reaction is shown in the Equation (8):

[nL-Fe^2+^(NO^+^,NO¯)] + ^•^NO + H_2_O → [nL-Fe^2+^(NO^+^,NO)] + HNO + OH¯ ↑↓         ↑↓ [nL-Fe^2+^(NO,NO)]   [nL-Fe^+^(NO^+^)_2_] (8)

where L are DNIC ligands, in our experiments—products of lysine interaction with MG.

The known DNICs have a g-factor of the EPR signal that ranges from 2.05 to 2.014 [39], e.g., for thiol- and phosphate-DNICs, the g-factor is close to 2.03. In the mixture containing only lysine or MG (Figure 7, spectra 1 and 4), the signal with a g-factor of 2.02 did not occur. In particular, when phosphate DNICs were added to the mixture with MG, only the EPR spectrum of the added complexes was registered (Figure 7, spectrum 1). The signal with g-factor 2.02 also disappeared almost completely in the presence of bathophenanthroline—the chelator of Fe_2_^+^, which destroys DNICs. Since the EPR signal of the new DNICs occurred in the first minutes after mixing the reaction mixture, AGEs cannot be ligands of these complexes. In DNICs, with histidine residues as ligands, iron is bound to the nitrogen of the imidazole ring [44]. Most likely, the iron of new-type DNICs is also coordinated with the nitrogen of Schiff bases and possibly the lysine carboxyl group. Indeed, since the EPR signal with a g-factor of 2.02 was not registered when lysine was replaced with Nα-acetyllysine, we can infer that α-amino group close to the carboxyl one is necessary for the formation of the ligand for DNICs of the new type.

**Figure 8:**
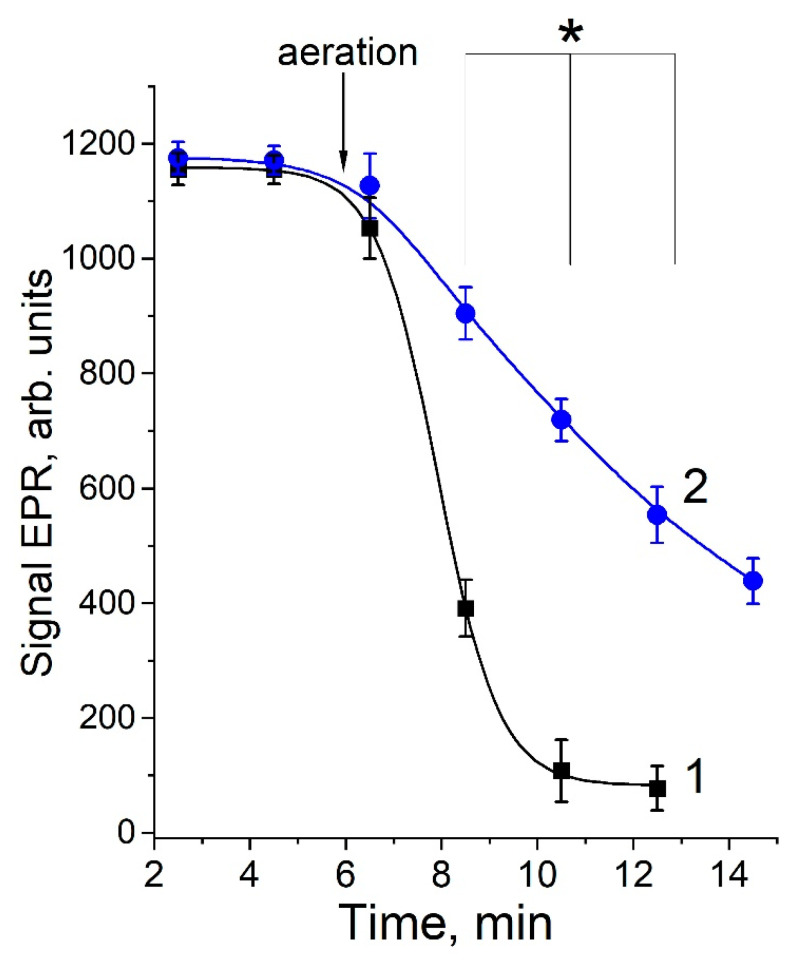
Kinetics of DNICs’ destruction associated with the products of lysine interacting with MG during aeration. The decomposition of the DNICs arising from mixing 160 mM lysine, 160 mM MG and 0.5 mM phosphate DNICs (1); in the presence of SOD (600 units/mL) (2). The reaction mixture was in a nitrogen atmosphere for the first 6 min, after which the incubation continued during aeration. Results are depicted as the mean with S.E.M. of three independent experiments (*n* = 3). Student’s *t*-test (* *p* ≤ 0.05 for all point after 8 min).

The DNICs containing modified lysine were rapidly destroyed under the aeration of the reaction mixture, while the rate of destruction decreased in the presence of superoxide dismutase (SOD) (Figure 8). This fact is consistent with our data on the intensive generation of O_2_^•−^ during the MG interaction with lysine [12].

DNICs with thiol ligands were previously shown to effectively intercept O_2_^•−^ and other ROS and RNS, which is associated with the antioxidant properties of DNICs [29,30,40,42,49]. It was assumed that in a reaction similar to reaction 1, between O_2_^•−^ and the NO-ligand of DNICs, peroxynitrite is formed, which remains bound to the iron of DNICs and is further isomerized to nitrate. The present research shows that the DNICs of the new type are able to intercept the superoxide radical formed at carbonyl stress modeling as well.

The antioxidant effect of DNICs may also arise from the chelatable iron included into the complexes [41,50]. In case of carbonyl stress, this should lead to inhibiting glycation and glycooxidation processes. It is known that the antiglycating activity of some compounds, e.g., pyridoxamine, is associated with the ability to chelate transition metal ions, and these compounds are considered to be potential pharmacological agents for diabetic hyperglycemia [46,51,52,53]. Thus, the formation of the new types of DNICs, associated with the products of the glycation of lysine, cysteine [43] or carnosine [44] by MG, may be an adaptation of the organism to an increase in ROS and RNS levels under carbonyl stress.

The discovered ability of ^•^NO-derived metabolites to influence the production and utilization of free radical intermediates of the Maillard reaction may play an important role in redox-dependent and electrophilic signaling [51,54]. In particular, these may be signaling pathways associated with nitroxyl anion and the free radicals of RSNOs [2,34].

### 2.3. The Effect of ^•^NO-Derived Metabolites on Non-Enzymatic Leghemoglobin Glycation

We have previously shown that GSNO and NO_2_^−^ inhibit the accumulation of the fluorescent products of metHb glycation by MG [4]. Hb-bound DNICs, which are associated with the cysteine of beta Hb subunit, can perform a similar function [29,30]. In addition, since, as we noted earlier, there is no cysteine in Lb, DNICs have to be bound to another amino acid residue.

One of the main targets of MG is known to be the lysine residues in protein molecules [6,8,11]. At the same time, it is the fluorescent products of non-enzymatic lysine glycation that intercept ^•^NO [20,55]. Therefore, we decided to study the effect of ^•^NO donors on non–enzymatic Lb glycation by MG.

Plant hemoglobins, including Lb, are important attendees in the crossroads between oxygen and ^•^NO [56]. They also participate in the metabolism of ^•^NO and its derivatives [57], and it was especially interesting to study Lb engagement in terms of the interaction of ^•^NO-derived metabolites and RCC.

So far, there have been no studies on the non-enzymatic glycation of Lb in nodules. We isolated the glycated Lb from *E. coli* cells with the plasmid-containing soy Lb*a* gene, grown at microaerophilic conditions [58]. The glycating Lb modification affects not only the protein surface, but also the heme pocket. This becomes evident after the changes in Lb spectral characteristics and the appearance of hemochromic type spectra [58]. It might stem from another feature in Lb structure—the presence of lysine in the heme pocket. This near-heme lysine is paired with glutamic acid and may be involved in the formation of AGEs, producing the sixth coordination bond with heme iron.

Lysine residues contribute to the formation of fluorescent cross-linked AGEs (pentosidine, pentodilysine, vesperlysine A, vesperlysine C, crossline, FPPC, AGE’XI) [59]. The main AGE structures of this group are imidazolium dilysine cross-links, also known as glyoxal–lysine dimer (GOLD) or MG–lysine dimer (MOLD) cross-links. Arginine and MG form a more fluorescent non-cross-linked AGE—argpyrimidine. Thanks to the autofluorescence of many AGEs, their formation on proteins can be registered by an increase in the fluorescence intensity at certain wavelengths (λ_ex_ = 320 − 370 nm, λ_em_ = 440 nm) [60].

**Figure 9:**
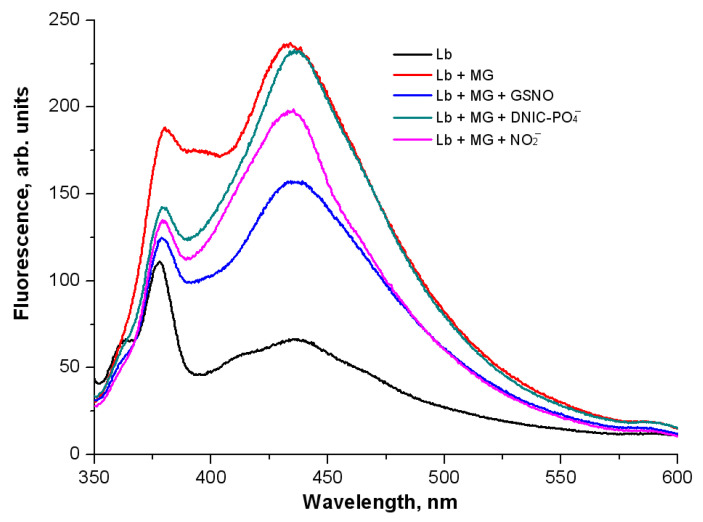
The effect of ^•^NO-derived metabolites on the formation of fluorescent products of non-enzymatic Lb glycation with MG. Reaction mixture: 0.1 mM metLb in 25 mM PBS (pH 7.4), 8 mM MG, and 2 mM of ^•^NO-derived metabolites (GSNO, DNIC-PO_4_^−^, NaNO_2_^−^).

Figure 9 shows the curves of the level of AGEs formed during the two days of bean Lb incubation with MG at 37 °C. The reaction took place under anaerobic conditions, which exclude peroxynitrite formation. Adding physiological NO donors (GSNO, DNIC-PO_4_^−^, NO_2_^−^) affected the fluorescence spectra of Lb-bound AGEs. DNIC-PO_4_^−^ had the least effect, while GSNO and NO_2_^−^ inhibited the formation of AGEs. GSNO reduced the fluorescence intensity by 40%, and NO_2_^−^ by 25%.

^•^NO can interact with non-enzymatic glycation products [6,20,55]. It was shown that the NO donors 2,2′-(hydroxynitrosohydrazono)bis-ethanamine (NOC18) and S-nitroso-N-acetyl-DL-penicillamine (SNAP) inhibited the formation of pentosidines [20]. The inhibitory effect is due to ^•^NO molecule, which is able to eliminate hydroxyl radicals, carbon-containing radicals and carbonyl compounds. In the model of carbonyl stress used, GS-DNICs (Section 2.2) and nitroxyl anions (Section 2.1) acted as antioxidants and anti-glycating agents.

DNIC-PO_4_^−^ molecules are donors not only of ^•^NO, but also of Fe^2+^, which is known to catalyze glycooxidation and AGE formation [60]. As a matter of fact, DNIC-PO_4_^−^, being a donor of Fe-(NO)_2_, could well participate in the formation of Lb-bound DNICs, as we have proven earlier [61]. Since Lb does not contain cysteines, it is likely that histidine residues are the ligands of these DNICs [44].

## 3. Discussion

Reactive small molecules—ROS, RNS and RCC—are inevitable companions of various metabolic processes in animal as well as plant organisms. These molecules directly react with free amino acids and amino acid residues in proteins. They also target lipids, nucleic acids, and sugars. As a result of these reactions, new reactive compounds are formed. In addition, ROS, RNS and RCC interact with each other. Collectively, all the reactions of reactive small molecules form a complex network of non-enzymatic interactions that regulate the development of an adaptive response of a living system to stress [9].

In our experiments with Lb, the anti-glycating effect may be due to the action of ^•^NO formed during the decay of GSNO or the reduction of NO_2_^−^. It should be noted that GSNO had an anti-glycating effect at the late stage of the reaction, when AGEs appeared. At the early stage, when Schiff bases had been formed, GSNO increased the yield of organic free radicals produced in the MG reaction with lysine (Section 2.1).

Thus, the results obtained show that ^•^NO donors and RSNOs affect the reaction of non-enzymatic protein glycation. In this case, both the slowing down of this reaction and a change in the set of products, e.g., the appearance of nitro- and nitroso-derivatives, can transpire.

Lysine is the amino acid most susceptible to non-enzymatic glycation [59,62]. Therefore, we used it in the studies on modeling the Maillard reaction in vitro. We also used Nα-acetyllysine as an analog of the amino acid residue in proteins and peptides, although it is clear that the data obtained, even on the modified free amino acids, cannot fully account for the processes with amino acid residues in proteins. The susceptibility of the lysine residue to glycation is affected by microenvironment. For example, the low-polar microenvironment of lysine in recombinant monoclonal antibodies (mAt) reduces the pKa of the ε-amino group of HC-CDR3-Lys98, which makes this amino acid extremely susceptible to glycation [62]. Studying the role of ^•^NO-derived metabolites in a simple system (amino acid and MG) simulating the action of non-enzymatic glycation is the first step in understanding the processes that develop with proteins under carbonyl stress.

As it is mentioned above, Lbs are lysine-rich proteins, while the lysine function in Lb has not been specially discussed in literature. It can be assumed that these lysine residues are “the glycation hot spots”, which regulate the function and lifespan of the protein. The existence of such hot spots has recently been shown in the plant and rhizobial proteomes of the common bean (*Phaseolus vulgaris*) root nodules [28].

However, the glycation of Lb in nitrogen-fixing nodules has not yet been investigated. This might arise from the fact that non-enzymatic glycation in plants has been studied much less than in animals. This is quite peculiar, since plants contain high concentrations of carbohydrates. A study of the plant proteome revealed that plant proteins are much more glycated than the animal ones [63,64]. The main glycating agents in plants are α-dicarbonyls (glyoxal and MG). Although the constitutive MG concentrations in plant cells are quite low, the amount of this metabolite significantly increases under environmental stresses (drought, flooding, bright light, salinization) [63,64]. The concentration of α-dicarbonyls usually becomes higher in the places where reducing sugars are accumulated and ROS production is increased, for example, in chloroplasts and mitochondria. It was suggested that non-enzymatic glycation and glycoxidation in plants have a physiological function [63,64]. On the one hand, AGEs can serve as markers for protein degradation. Meanwhile, reactive sugars and ketoaldehydes can participate in sugar signaling, adding to the complexity of the issue [63]. It has to be noted that in the article [28], the Lb carbonylation in nodules was reported that led to the aggregation of Lb molecules, but not the Lb glycation.

It is possible that the primary function of lysines in Lb was to provide high stability and solubility of the protein molecule [65,66,67,68]. Lysine is a positively charged basic amino acid, and its residues are often found on the surface of proteins. Lysine has a high pKa value, which allows it to form stable ionic and electrostatic interactions [68] and to increase protein solubility. This fact is applied to the myoglobin (Mb) of diving mammals, a lysine-rich protein. This Mb has a large positive charge that causes electrostatic repulsion of its molecules, which maintains Mb solubility at high concentrations in myocytes [69]. Moreover, lysine residues may increase the solubility of Lb, whose concentration in the cytosol of plant cell is also very high (~40% of all soluble proteins) [70,71].

Lysine residues undergo posttranslational modifications (methylation, acetylation, glycation, etc.), which significantly affect the protein’s properties and its tendency for aggregation. For example, the acetylation of apomyoglobin led to the formation of amorphous aggregates [66]. The study of Mb glycated with glucose, D-ribose, 3-deoxyglucosone and MG showed that in this protein, AGEs were formed on lysine and arginine residues. In the case of Mb reacted with MG, over half of lysine residues underwent modification [72], forming Nε-carboxyethyl lysine (CEL) and MG-derived hydroimidazolone-1 (MG-H1). The glycation of lysine residues leads to the formation of bis(lysyl)imidazolium cross-links and neutralization of positive charges [72,73]. This is the reason for the formation of Mb aggregates, which may be of an amyloid nature [73]. Since Lb is a protein structurally similar to Mb, we can expect similar consequences of its reaction with MG.

Glycation of Lb lysines can occur both in the natural Lb environment (in nodule plant cells) and in the expression system (bacteria, yeasts). We suppose that this can lead to a decrease in the solubility of Lb (Mb-like protein) and the formation of covalently cross-linked aggregates, which can affect the effectiveness of nitrogen fixation in legume nodules, and the efficiency of recombinant Lb synthesis in microorganisms–producers. It can also have a negative effect on the viability of these microorganisms [61]. Therefore, a better understanding of the factors that affect the glycation of lysines and lysine-rich proteins can be used to increase the stability and solubility of Lb. Revealing the mechanisms of these processes can help us to understand the influence of various stress factors on legume plants and on microorganisms–producers used in biotechnology.

Thus, Lb glycation was directly shown on recombinant protein, and glycated Lb was isolated from *E. coli* cells [58]. We could obtain glycated Lb in the model system with isolated nodule Lb. The existence of this process in legume nodules can still be judged by indirect signs, and has to be further studied.

## 4. Materials and Methods

### 4.1. Materials

The following reagents were used in the present work: N*α*-acetyl-*L*-lysine (*Nα*-acetyllysine), bathophenanthroline, *L*-cysteine, diethylenetriaminepentaacetic acid (DTPA), *L*-glutathione reduced (GSH), lysine (*L*-lysine was always used), MG solution, FeSO_4_, KH_2_PO_4_, Na_2_HPO_4_, NaNO_2_, NaOH, phenylmethylsulfonyl fluoride (PMSF), superoxide dismutase from bovine erythrocytes—“Sigma-Aldrich” (St. Louis, MO, USA); 4-hydroxy-(2,2,6,6-tetramethylpiperidin-1-yl)oxyl (4-hydroxy-TEMPO)—“Oxis” (Portland, OR, USA); Z)-1-[N-(3-aminopropyl)-N-(n-propyl)amino]diazen-1-ium-1,2-diolate (PAPA NONOate), nitroxyl donor Sodium trioxidinitrate (Angeli’s salt)—“Cayman Europe” (Tallinn, Estonia); gas-permeable PTFE Sub-Lite-Wall tubing—“Zeus Industrial Products” (Orangeburg, SC, USA).

### 4.2. The Synthesis of RSNOs and DNICs

GSNO and CysNO were obtained by mixing equimolar concentrations of thiols (GSH or cysteine) and sodium nitrite (NaNO_2_) in an acidic medium as described in the articles [4,29,31]. The reaction mixture was incubated for 15 min in the dark at room temperature (~20 °C). The RSNO concentration was determined spectrophotometrically by optical absorption at 338 nm for GSNO (ε_338_ = 930 M^−1^cm^−1^) [4] and 335 nm for CysNO (ε_335_ = 922 M^−1^cm^−1^) [40]. UV-visible absorption spectra were recorded on Saga 300 UV-VIS spectrophotometer (“VarianBio”, Palo Alto, CA, USA) at room temperature in a 1 cm optical cuvette at scanning speed 600 nm/min.

DNICs with phosphate ligands were obtained by passing gaseous NO through a FeSO_4_ solution (5.5 mM) in 100 mM K,Na-phosphate buffer (pH 6.8) in the Thunberg vessel. To obtain GSH- and cysteine-containing DNICs, the corresponding thiols were added to phosphate DNICs in a molar ratio of 1:2.25. The shift of the equilibrium of thiol-containing DNICs towards paramagnetic mononuclear form was achieved by the addition of cysteine to a 1:20 molar ratio. The DNICs concentration was determined based on the overall intensity of the EPR signal of the complexes, with 4-hydroxy-TEMPO spin label as an external standard. The RSNOs and DNICs preparations were stored at liquid nitrogen temperature (−195.8 °C).

### 4.3. EPR Measurements

EPR spectra were recorded and analyzed on E-109E spectrometer (“Varian”, Palo Alto, CA, USA). The reaction mixture was drawn into a gas-permeable PTFE Sub-Lite-Wall tubing (inside diameter 0.625 mm, wall thickness 0.051 mm) (“Zeus Industrial Products”, Orangeburg, SC, USA). This capillary tubing was folded twice and inserted into a quartz EPR tube open at both ends, allowing adequate gas flow (N_2_ or mixture of O_2_ and N_2_) around the sample in the TE102 EPR cavity. EPR spectra were simulated by SimFonia software (“Bruker”, Karlsruhe, Germany). Recording settings were as follows: microwave power 20 mW, high-frequency modulation amplitude 0.1–0.4 mT, microwave frequency 9.15 GHz, scan time 120 s, temperature 25 °C. The EPR signals of the stable synthetic free radical diphenylpicrylhydrazine and the spin label hydroxy-TEMPO were used as a standard.

### 4.4. The Isolation and Purification of Lb from Bean Nodules

Lb was isolated from the root nodules of beans (*Vicia faba* L., synonym—*Faba bona* Medik.), the cultivar “Russian black”, which were collected at the stage of budding—beginning of flowering. Detached nodules were filled with 0.1 M PBS (pH 7.2) containing 0.2 M sodium ascorbate, 1% high MW polyvinylpyrrolidone, 50 µM PMSF and 1 mM MgCl_2_, in ratio buffer:nodules—1.5:1 by weight. The nodules were rubbed in agate mortar, and the homogenate was filtered. The filtrate was centrifuged at 1000× *g* for 5 min to remove the large particles of plant tissue. The Lb-containing fraction was obtained by salting proteins with ammonium sulfate in a 40–80% saturation range. The precipitate was dissolved in 0.02 M PBS (pH 7.2), and centrifuged at 23,000× *g* for 20 min. The supernatant was applied to a Toyopearl HW-50 Fine (“Toyo Soda”, Tokyo, Japan) column (43 × 2.2 cm) in 25 mM Tris–HCl buffer (pH 7.5) with 0.2 M NaCl. To suppress the activity of proteases, 10 µM of PMSF was added. The next stage of Lb purification was anion exchange chromatography on a Servacel DEAE GS (“Serva”, Heidelberg, Germany) column (7 × 2 cm), with 0.01 M PBS (pH 7.2). Lb fractionation was carried out with a linear NaCl gradient from 0 to 1 M. The Lb fraction was eluted in the range of 0.2–0.4 M NaCl. The eluate was oxidized with potassium ferricyanide and dialized in 25 mM PBS (pH 7.2). All procedures were carried out at 4 °C. Lb concentration was measured with the modified pyridine hemochromogen method [61].

### 4.5. The Non-Enzymatic Glycation of Leghemoglobin

MG was used as an agent for non-enzymatic Lb glycation. The reaction medium contained 0.1 mM metLb in 25 mM PBS (pH 7.4), 8 mM MG, and 2 mM ^•^NO-derived metabolite (GSNO, phosphate DNICs or NaNO_2_). ^•^NO-derived metabolites were added after the 20-min Lb incubation with MG. The vials were purged with gaseous nitrogen for 15 min. The incubation was carried out at 37 °C for 48 h. The final products of non-enzymatic Lb glycation were measured by autofluorescence at λ_ex_ = 335 nm. Before measurement, the samples were diluted in a ratio of 1 to 12. The measurement was carried out on Shimadzu RF-5302 PC spectrophotometer (“Shimadzu”, Kyoto, Japan) in a 0.5 mL micro-cuvette at a medium scanning speed, with high sensitivity (according to the unit designation). The slit width of excitation and emitting light were 5 nm and 10 nm, respectively.

### 4.6. Statistical Analysis

The measurements were performed in at least three replicates for each sample. The statistical data were processed based on 3-4 analytical repetitions. The data are presented as a mean ± standard deviation. The ANOVA statistical model and Student’s *t*-test were used for the obtained data analysis.

## 5. Conclusions

The obtained results indicate a close connection between the processes occurring under carbonyl, oxidative and nitrosative stresses yielding a complex regulative network of non-enzymatic interactions. ^•^NO metabolites can regulate the non-enzymatic generation of free radicals arising in the MG reaction with amino acids. RSNOs stimulate the organic free radicals to be formed, whereas DNICs and nitroxyl anions, on the contrary, decrease their levels. At the same time, the DNICs associated with the products of the MG reaction with lysine are able to intercept superoxide, which is also formed there. We suppose that the formation of certain ^•^NO-derived metabolites under carbonyl stress can change both the amount of free radical intermediates of the Maillard reaction and the quantity and composition of the final products of non-enzymatic glycation. The discovered effects of NO metabolites can be observed not only in living systems, but also in food products, where the non-enzymatic glycation occurs as well.

The data on the effect of NO metabolites on the glycation of lysines by MG can help to explain a similar situation with Lb—the symbiotic hemoglobin of legume plants. In particular, ^•^NO and GSNO slow down the formation of fluorescent AGEs in Lb. Further studies of the Lb glycation process can help in better understanding the impact of stress factors on legume plants and contribute to the production of recombinant Lb for biotechnology.

## Data Availability

The data presented in this study are available in the article.
